# *Staphylococcus aureus* Putative Vaccines Based on the Virulence Factors: A Mini-Review

**DOI:** 10.3389/fmicb.2021.704247

**Published:** 2021-09-03

**Authors:** Bahman Mirzaei, Ryhaneh Babaei, Habib Zeighami, Maryam Dadar, Ali Soltani

**Affiliations:** ^1^Department of Medical Microbiology and Virology, School of Medicine, Zanjan University of Medical Sciences, Zanjan, Iran; ^2^Razi Vaccine and Serum Research Institute, Agricultural Research, Education and Extension Organization, Karaj, Iran; ^3^Department of English Language, Zanjan University of Medical Sciences, Zanjan, Iran

**Keywords:** candidate vaccines, immunoprophylaxis, prophylaxis, *Staphylococcus aureus*, staphylococcal infections *Staphylococcus aureus*

## Abstract

Since the 1960s, the frequency of methicillin*-*resistant *Staphylococcus aureus* as a recurrent cause of nosocomial infections has increased. Since multidrug-resistant *Staphylococcus* has overcome antimicrobial treatment, the development of putative vaccines based on virulence factors could be a great help in controlling the infections caused by bacteria and are actively being pursued in healthcare settings. This mini-review provides an overview of the recent progress in vaccine development, immunogenicity, and therapeutic features of some *S. aureus* macromolecules as putative vaccine candidates and their implications against human *S. aureus*-related infections. Based on the reviewed experiments, multivalent vaccines could prevent the promotion of the diseases caused by this bacterium and enhance the prevention chance of *S. aureus* infections.

## Highlights

- Vaccine development against staphylococcal infections is still in its infancy. Irrefutably, more studies on staphylococcal virulence factors and immune evasion are needed to reach a complete understanding of virulence mechanisms.
- Many investigations have put forward a large number of targets for vaccine development against *Staphylococcus aureus*, which increase the number of putative targets.
- Since numerous changeable infection-related factors exist and are also expressed in staphylococcal species, multivalent vaccines consisting of several antigens related to different infection stages are required.

## Introduction

*Staphylococcus aureus* is a widespread commensal and pathogen bacterium. *S. aureus* bacteria induce staph food poisoning that leads to gastrointestinal illness through eating foods contaminated with the toxins produced. About 25% of animals and people have staph in their nose and on their skin (Le Loir et al., 2003). It is also one of the most isolated bacteria among both nosocomial and community-acquired infections. It causes many types of human infections and syndromes such as mild skin and soft tissue infections, bacteremia, endocarditis, pneumonia, metastatic infections, sepsis, and toxic shock syndrome (van Belkum, 2006). A hospital environment and medical devices contaminated with *S. aureus* can affect the health of patients. Over the past decades, staphylococcus nosocomial infections have significantly increased (Kuklin et al., 2006; Hogea et al., 2014). Since the 1960s when the first methicillin-resistant *S. aureus* (MRSA) was identified, a major challenge has begun (Adhikari et al., 2012). The emergence of antibiotic-resistant strains of staphylococci, mainly MRSA, emphasizes the serious control of *S. aureus*-related infections (O’Neill et al., 2008)—for example, the outbreak of *S. aureus* bloodstream infections in the United States in 2017 induced nearly 20,000 deaths (Kourtis et al., 2019). However, there is no current vaccine for *S. aureus* infection. Several *S. aureus* virulence factors have been evaluated as vaccine candidates. Infections caused by MRSA in hospital wards have decreased due to increased health assessments and the presentation of effective vaccines. *Staphylococcus* spp. conserved surface components with a high rate of expression in the bloodstream or biofilm-forming process factors stand as suitable staphylococcal candidate vaccines to decrease the staphylococcal disorders (Van Mellaert et al., 2012; Hogea et al., 2014). Thus, it is essential to know the relevant factors involved in biofilm formation from a molecular pathogenesis perspective and to discover the physiological status of these virulence factors within the body in order to realize whether they have the potency to develop an aggressive behavior.

## Discussion

### Vaccine Development Based on the Targets

Many investigations have put forward a large number of targets for vaccine development against *S. aureus*, which increase the number of putative targets. In the classical approach, different targets with certain functions have been studied and evaluated as subunit vaccine. New target candidates have also been suggested by reverse vaccinology and bioinformatics (Zhang et al., 2003; Bowden et al., 2005; Gill et al., 2005). In order to cover the genetic diversity of a pathogen in vaccine development strategies, its pan-genome should be analyzed, and its molecular epidemiology should also be examined (Mora and Telford, 2010).

### The Search for Vaccine Targets

Poly(glutamic acid) (PGA) stands for a good vaccine candidate against the mentioned bacterium, owing to its protection effects against antimicrobial peptides during biofilm-related infections and neutrophil phagocytosis. The result of an experiment indicated that arisen antibodies to conjugated PGA are able to protect three models of animals, including guinea pig, mouse, and rabbit, against anthrax (Joyce et al., 2006).

Phenol-soluble modulins (PSMs) are considered as another promising group as vaccine target. Recently, a study showed that PSMβ peptides had an inhibitory effect on bacterial dissemination from implants (Rennermalm et al., 2004; Wang et al., 2011). Unlike most mentioned vaccine candidates, PSMβ interferes the dissemination of biofilm-associated infection *via* preventing detachment mechanisms.

## Some Putative Vaccine Candidates to *S. aureus*

### Capsular Polysaccharide

The function of conjugated microencapsulated *S. aureus* type 8 (the isolate came from bovine mastitis milk) to *Pseudomonas aeruginosa* exotoxin A (ETA) was assessed in a mouse model. The antibody response was triggered 3 days following the immunization and lasted for 13 days of the observation period after the second injection in some mice. The antibody response and the survival rate were higher in the group of mice immunized with the CP8–ETA conjugates in comparison with those receiving complete Freund’s adjuvant or phosphate-buffered saline. Based on the result of this experiment, the CP8–ETA vaccine is able to protect mice against *S. aureus* bacteremia (Han et al., 2000).

### Iron-Regulated Surface Determinant B

The *S. aureus* iron-regulated surface determinant B (IsdB), a prophylactic vaccine against *S. aureus* infection, as an iron-sequestering protein exists in many *S. aureus* clinical isolates and methicillin-resistant and methicillin-sensitive isolates and is expressed on the surface of all tested isolates. As the mice were immunized with IsdB formulated with amorphous aluminum hydroxyphosphate sulfate, high immunogenicity of IsdB in rhesus macaques was observed. Furthermore, a fivefold increase in antibody titers was seen after a single immunization, which indicates IsdB potency as a vaccine against *S. aureus* disease in humans (Jones et al., 2001; Kuklin et al., 2006). A randomized study on the preoperative receipt of Merck V710 *S. aureus* vaccine containing non-adjuvanted IsdB demonstrated that all V710 recipients and only about 8% of the placebo recipients died of postoperative *S. aureus* infection following a major cardiothoracic surgery. These results may raise the concern of researchers about the immunization itself, which might affect either the safety or the efficacy of the development of staphylococcal vaccines (McNeely et al., 2014; Daly et al., 2017). In another cohort study, in spite of modern perioperative management, postoperative *S. aureus* infection occurred in 1% of adult patients. The mortality rates were also 3% for methicillin*-*resistant *S. aureus* infections and 13% for MRSA infections (Allen et al., 2014).

### Virus-Like Particle-Based Vaccines

The coordination of the expression of the required virulence factors in the invasive infection of *S. aureus* happens using secreted cyclic auto-inducing peptides (AIPs) and the accessory gene regulator (*agr*) operon. AIPs are small in size and require a thiolactone bond. In order to solve this issue, the virus-like particles were utilized as a vaccine platform (PP7) for a conformationally restricted presentation of a modified AIP1 amino acid sequence (AIP1S). AIP1-specific antibodies inhibited *agr* activation *in vivo*; moreover, it reduced pathogenesis and increased bacterial clearance in murine skin and a soft tissue infection model carrying a highly virulent *agr* type I *S. aureus* isolate, which all indicated vaccine efficacy and that it might have a great impact on antibiotic resistance (Daly et al., 2017).

### *Staphylococcus aureus* Alpha-Hemolysin

Based on the results of previous studies, a recombinant vaccine for *S. aureus* alpha-hemolysin should have a heptameric structure for its crystal. HIa, a pore-forming toxin, is expressed by the majority of *S. aureus* strains. HIa was examined for vaccination with AT-62aa along with a glucopyranosyl lipid adjuvant–stable emulsion. Then, the results indicated that sepsis protection in an experimental model of *S. aureus* infection was done by utilizing Newman and the pandemic strain USA300 (LAC). This model demonstrated the AT-62aa is a proper vaccine candidate. The identification of AT-62aa protective epitopes may also result in novel immunotherapy for *S. aureus* infection (Adhikari et al., 2012).

### *Staphylococcus aureus* LukS-PV-Attenuated Subunit Vaccine

LukS-mut9 is an attenuated mutant of LukS-PV with a high immunogenic response. This mutant has shown significant protection in mouse sepsis model. Recent findings revealed that the protection of the Panton–Valentine leukocidin (PVL) vaccine in mice model is related tocross-protective responses against other homologous toxins, owing to the generated polyclonal antibodies by LukS-mut9, which can neutralize other canonical and non-canonical leukotoxin pairs. There has been a correlation between the arisen antibodies, PVL subunits, and sepsis in patients with high antibody titer against the mentioned subunits (Adhikari et al., 2012).

### Four-Component *Staphylococcus aureus* Vaccine

In a study conducted based on a murine *S. aureus* infection model, antigen-specific antibodies were accumulated in the pouch, and the infection was mitigated following immunization with 4CStaph and bacterial inoculation in an air pouch generated on the back of the animal. The upregulation of FcR and the presence of antigen-specific antibodies induced by immunization with 4CStaph could increase bacterial opsonophagocytosis. Alternative protection mechanisms may be activated by a proper vaccine, balancing neutropenia, which is a condition often happening to *S. aureus*-infected patients (Torre et al., 2015).

### The Mixture of PBP2a and Autolysin as a Candidate Vaccine Against Methicillin-Resistant *S. aureus*

**FIGURE 1:**
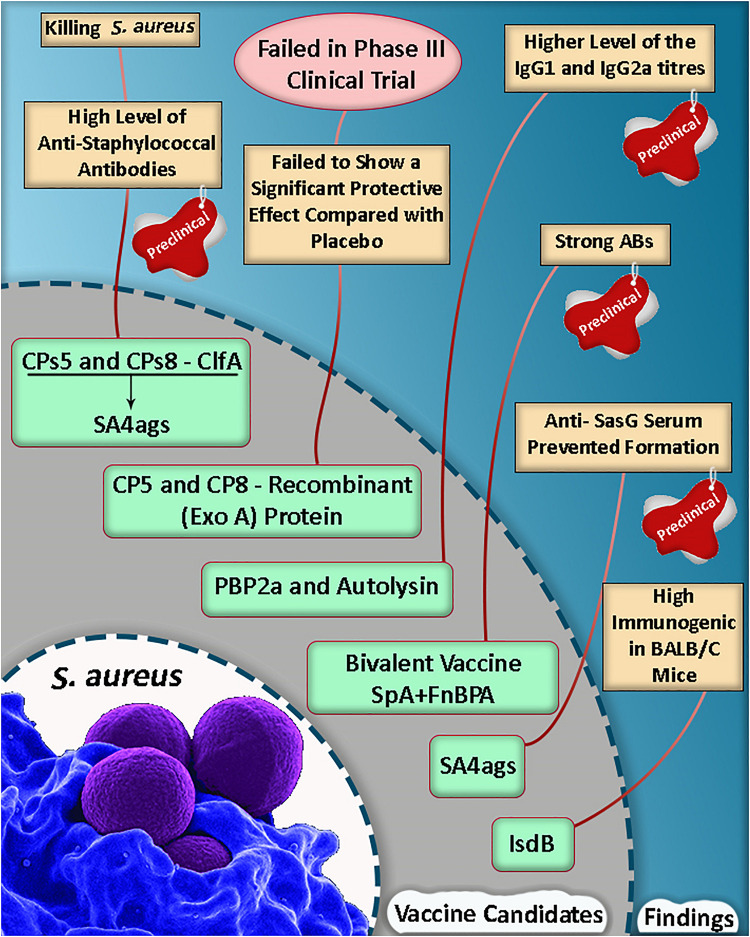
A summary of vaccine candidate development in *Staphylococcus aureus* (Shinefield et al., 2002; Kuklin et al., 2006; Fowler et al., 2013; Haghighat et al., 2017; Dupont et al., 2018; Yang et al., 2018).

Based on a study, the mortality rate was reduced in mice, and they were protected against lethal MRSA challenge as well as single proteins following an active vaccination with a mixture of r-PBP2a/r-autolysin and a conjugated form of the vaccine (Haghighat et al., 2017). Some of the selective putative vaccine candidates and a summary of the vaccine candidate development in *S. aureus* are listed in Table 1 and Figure 1.

**TABLE 1:** Some putative vaccine candidates which could be considered in vaccine development against *Staphylococcus aureus*.

**Putative macromolecules**	**Features**	**Advantage**	**Disadvantage**	**References**
Polysaccharide intercellular adhesion (PIA)	Surface polysaccharide poly-N-acetyl-β-(1-6)-glucosamine also known as PIA	Produced *in vitro* by either *S. aureus* or *Staphylococcus epidermidis* with high levels of acetate substituting for amino groups; generate opsonic and protective antibodies PIA has been extensively evaluated as a putative candidate for vaccine development	Immunization with PIA and other polysaccharides must be boosted or conjugated to a safe protein carrier	Maira-Litrán et al., 2005; Maira-Litrán et al., 2012; Miller et al., 2020
Teichoic acid	(A) Glycerol and ribitol phosphate copolymer by phosphodiester bonds (B) It is assigned as main macromolecule to the primary attachments and accumulation phase in biofilm formation (C) It is chiefly important in inflammation and immune evasion	One of the main Gram-positive bacteria-adhesive macromolecules In a study, the efficacy of mAb was determined as >90% against CoNS clinical isolates. Up to 90% of bacterial killing activity was detected at doses <10 μg/ml as an apt opsonophagocytic result, which prevents related infections in animal models	Immunization with PIA and other polysaccharides must be boosted	Ali et al., 2020; van Dalen et al., 2020
Accumulation-associated protein	Presence in both *S. aureus* and *S*. *epidermidis* that plays an essential role in the attachment and aggregation of biofilm phases	Polyclonal antibodies inhibit biofilm formation Its conjugation to a confirmed protective polysaccharide, such as PIA, could eliminate the biofilm formation process by inducing cellular immunity-related immunoglobulin subtypes (IgG2a and IgG2b) to activate memory cells	Arisen antibodies to AaP have no effect on polysaccharide-dependent biofilm-forming *S. aureus* and *S. epidermidis*	Yan et al., 2014
Fibronectin binding protein A	Presence in *S. aureus*	Specific antipeptide immunoglobulin (Ig) G and IgA antibodies were detected in the serum and respiratory mucosa of vaccinated mice. Responses to the major pilus backbone protein Spy0128 showed robust antibody responses to this antigen both systemically and in the respiratory and intestinal mucosa	The mechanism(s) of protection are unclear	Clow et al., 2020
Virulence factor	Secreted factors α-hemolysin, staphylococcal enterotoxin B, and the three surface proteins staphylococcal protein A, iron surface determinant B N2 domain, and manganese transport protein C	Induce comprehensive cellular and humoral immune responses to reduce bacterial loads, inflammatory cytokine expression, and inflammatory cell infiltration and decrease pathology after challenge with a sub-lethal dose of *S. aureus*	No significant differences in lymphocyte subset distribution and serous cytokine levels (IL-4, IL-5, TNF-α, IFN-γ, IL-2, and IL-6) between the vaccine and the placebo groups	Creech et al., 2020; Zeng et al., 2020; Alabdullah et al., 2021
Phosphatidylinositol phosphodiesterase	Secreted by extracellular pathogens such as *S. aureus*	Strong humoral response in the vaccine mice that provided 75% protection against *S. aureus*	Large-scale *in vivo* studies are called	Soltan et al., 2020

*AaP, accumulation-associated protein; TA, teichoic acid; mAB, monoclonal antibody.*

## Limitation

Several vaccine candidates which are of recent progress in vaccine development are only presented in this study. Therefore, more explanation was not mentioned about the general function of the vaccine candidate molecules, particularly with regard to PSM, ETA, IsdB, alpha-hemolysin, LukS-PV, PBP2a, and autolysin.

## Conclusion

Vaccine development against staphylococcal infections is still in its infancy. Irrefutably, more studies on staphylococcal virulence factors and immune evasion are required to enable us to reach a complete understanding of the virulence mechanisms. Since numerous changeable infection-related factors exist and also expressed in staphylococcal species, multivalent vaccines consisting of several antigens related to different infection stages are needed. There are few ways to deal with *S. aureus* infections due to their high antibiotic resistance and also because the infections caused by this microorganism are increasing. However, fortunately, since sufficient research has been done on the effects of various vaccine candidates regarding the *S. aureus* virulence factor, the capability of biofilm production could be noticed as one of the most important factors in bacterium colonization as well. If a suitable vaccine candidate can be included to (1) inhibit biofilm formation and (2) prevent the effect of bacterial virulence factors, then the possibility of preventing and eliminating infections can be imagined. It is expected that designing a multivalent vaccine with the above-mentioned content will raise the effectiveness of antibodies and lead to the eradication of *S. aureus*-related infections.

## Author Contributions

BM contributed to conceptualization, data collection, data curation, and writing of the manuscript. RB, HZ, and MD contributed to data collection. AS contributed to data collection and writing of the manuscript. All authors read and approved the manuscript.

## Conflict of Interest

The authors declare that the research was conducted in the absence of any commercial or financial relationships that could be construed as a potential conflict of interest.

## Publisher’s Note

All claims expressed in this article are solely those of the authors and do not necessarily represent those of their affiliated organizations, or those of the publisher, the editors and the reviewers. Any product that may be evaluated in this article, or claim that may be made by its manufacturer, is not guaranteed or endorsed by the publisher.
